# Contributions of Australian University Departments of Rural Health to Indigenous Health Intervention Research: A Narrative Review

**DOI:** 10.3390/healthcare14050595

**Published:** 2026-02-27

**Authors:** Samantha Bay, Katrina P. Fyfe, Annette McVicar, Emma Walke, Charmaine Green, Emma V. Taylor, Ha Hoang, Lisa Hall, Carrie Lethborg, Sandra C. Thompson

**Affiliations:** 1 Western Australian Centre for Rural Health, University of Western Australia, 167 Fitzgerald Street, Geraldton, WA 6530, Australia; samantha.bay@uwa.edu.au (S.B.); sandra.thompson@uwa.edu.au (S.C.T.); 2 Western Australian Centre for Rural Health (Pilbara), University of Western Australia, 66 Welcome Road, Karratha, WA 6714, Australia; 3 University Centre for Rural Health, University of Sydney, 61 Uralba Street, Lismore, NSW 2480, Australia; 4 Centre for Rural Health, University of Tasmania, Locked Bag 1362, Launceston, TAS 7250, Australia; 5 Monash Rural Health Bendigo, Monash University, 26 Mercy St, Bendigo, VIC 3552, Australia; 6 Social Work Department, St Vincent’s Hospital, 41 Victoria Parade, Fitzroy, VIC 3065, Australia; 7 Department of Medicine, University of Melbourne, Grattan Street, Parkville, VIC 3010, Australia

**Keywords:** Aboriginal and Torres Strait Islander, First Nations, Indigenous, intervention, health services research, rural health workforce, rural health academic centre, rural research, university department of rural health

## Abstract

**Highlights:**

**What are the main findings?**

- Successful interventions often use multicomponent approaches, combining systemic changes with individual-level supports to address complex health issues.
- Sustainability of interventions is associated with adequate resourcing, systemic embedding of programs, and clear pathways for ongoing care. Strong organisational leadership and collaboration are essential to embed interventions into healthcare systems, increase capacity building, and sustain long-term impact.

**What are the implications of the main findings?**

- Effective interventions in Indigenous health require meaningful community engagement to ensure alignment with needs, priorities and cultural values. Culturally safe interventions should include Indigenous stakeholders in co-design processes, with leaders, staff and students supported by cultural safety training and experiential learning opportunities.
- Targeting underlying determinants of health such as economic, social, psychological and environmental factors fosters participation and achieves sustainable outcomes. Interventions must address barriers like access to services, resources, and literacy levels.

**Abstract:**

**Background/Objectives:** University Department of Rural Health (UDRH) programs were created to address the disparities in rural Australian communities. A large proportion of Aboriginal and Torres Strait Islander people live in rural communities, and the UDRHs work closely with communities to improve outcomes for Indigenous Australians. This narrative review examines the nature of the intervention papers in Australian Indigenous health published by UDRHs and identifies key learnings to improve interventions in Australian Indigenous health. **Methods:** Intervention papers were identified from an established database of UDRH Indigenous health-related publications published 2010–2021. **Results:** Thirty-three papers were included in the review. Thematic analysis identified four overarching themes from the key learnings identified in the papers: (1) principles of engagement and design; (2) considerations for improving healthcare systems; (3) considerations for improving healthcare workforce; and (4) the sustainability of interventions and improvements in outcomes. Most of the studies employed qualitative or mixed-methods designs. **Conclusions:** These findings provide practical guidance for strengthening Indigenous health interventions. Effective Indigenous health interventions require meaningful community engagement and co-design, culturally safe practice supported by workforce training, and multicomponent approaches that address social determinants and barriers to access. Sustained impact depends on adequate resourcing, strong organisation leadership and embedding programs within healthcare systems with clear pathways for ongoing care and capacity building. UDRHs should reflect on current and future projects to ensure that engagement principles, system-level considerations, health workforce development, and long-term sustainability are embedded within intervention design and implementation.

## 1. Introduction

Australia has a large geographic area with a substantial concentration of the population living in major cities [1]. Hence, it is unsurprising that resources and research have been biased toward metropolitan populations [2,3]. Compared to those living in metropolitan areas, people in rural and remote Australia, including Aboriginal and Torres Strait Islander people (henceforth respectfully referred to as Indigenous), have poorer life expectancy, are at higher risks of illness and injury, and experience disadvantages associated with social determinants such as access to healthcare services and education [1]. There are also well-described challenges with sustaining a health workforce in rural and remote areas to provide services in regions with small populations [4,5].

In response to the disparities and challenges in rural health, successive Australian governments have invested in University Department of Rural Health (UDRH) programs since the late 1990s to increase the number of allied health and nursing in rural and remote locations and to develop pathways for Indigenous people to complete education in health sciences degrees and research. Given UDRHs’ locations in rural areas with substantial Indigenous populations, UDRHs often engage closely with local Indigenous communities and Aboriginal Community Controlled Health Organisations to develop teaching and learning opportunities. The Australian Rural Health and Education Network (ARHEN) provides a structure under which the UDRHs share information and collaborate around common aims of improving the health and well-being of rural communities. Given their focus on reducing health disparities and the higher proportion of Indigenous Australians with increasing remoteness, it is unsurprising that UDRHs have been engaged in research around Indigenous health [6,7]. In 2019 ARHEN established a database to serve as a central repository of UDRH health, training and workforce research conducted since 2010.

Sanson-Fisher and colleagues [8] and Thompson and colleagues [9] identified that the bulk of research about Indigenous people has been descriptive in nature, with a small proportion describing interventions. Without dismissing the importance of descriptive research, there is need to move from describing and measuring health issues to implementing and evaluating interventions to target these health issues. Importantly, interventions in Indigenous health must be underpinned by principles of cultural safety and cultural security. Cultural safety is “about how care is provided, rather than what care is provided” [10] and requires healthcare practitioners and researchers to critically reflect on power imbalances, with cultural safety determined by Indigenous individuals and communities [11,12]. Cultural security refers to the systems, policies and procedures that actively ensure services are delivered in ways that are respectful, relevant and responsive to Indigenous peoples sustainably [11]. Together, these concepts emphasis Indigenous leadership, community governance and accountability as central to ethical and effective health interventions.

A previous review explored researcher-reported strengths and limitations of Indigenous health research intervention and evaluation trials and concluded that leadership of Indigenous researchers and communities, adequate resourcing, flexible and long timeframes for projects are enablers for appropriate and ethical Indigenous health interventions [13]. Our review explored beyond researcher-reported strengths and limitations, by deriving key learnings from qualitative and quantitative research relating to interventions in Indigenous health, including interventions for communities, patients, healthcare workers, services, and health systems. This study was conducted as part of a larger project to identify and describe the contributions of UDRHs to Indigenous issues through analysis of the ARHEN database during the period 2010–2021. We explored the intervention studies identified in Thompson and colleagues’ paper [9]. We aimed to examine the nature of the intervention papers in Australian Indigenous health published by UDRHs and make suggestions from the key learnings to improve health services and systems.

## 2. Methods

A research team of ten people from four UDRHs was formed in 2024. The research team consisted of three Indigenous researchers: AM, a Mununjahli and Minjungbal woman with a background in education and training, EW, a Bundjalung woman and experienced researcher, and CG, a Wajarri, Badimaya and Wilunyu woman and experienced researcher. The non-Indigenous members of the research team have extensive experience in Indigenous health research, cultural education and training, and rural health.

### 2.1. Identification and Screening

This narrative review is embedded within a larger project exploring UDRH publications in Indigenous health. This narrative review analysed Indigenous health intervention papers that were previously classified as intervention research by Thompson and colleagues [9]. The previous study identified 493 publications sourced from the ARHEN database. Included articles examined Indigenous health issues, were published between 2010 and 2021, and included at least one author affiliated with a UDRH. A detailed description of the search strategy and screening process used to identify these publications is available in the previous study [9].

In the previous study, publications were categorised as descriptive, interventions, and measurement [9]. Publications were classified as interventions if they involved clinical or public health interventions or if they aimed to influence health-related knowledge, attitudes or behaviours or improve healthcare delivery. Of the 493 publications identified, 36 met the criteria for intervention studies. Thompson et al. [9] recommended exploration of these publications in greater detail, which therefore constituted the focus of the present narrative review.

**Figure 1:**
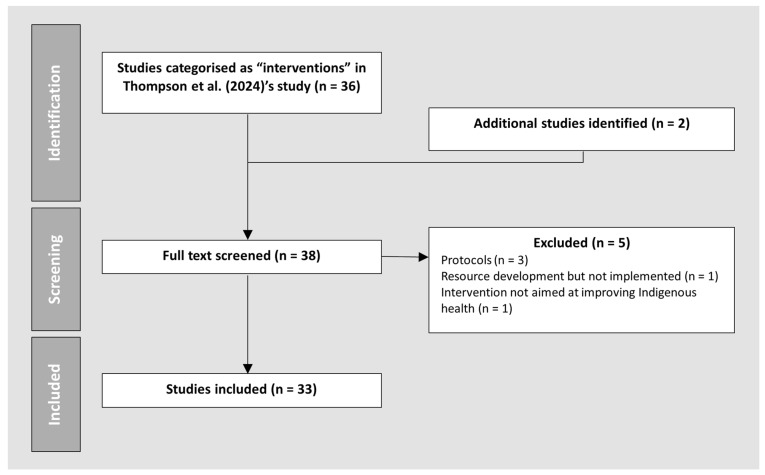
Process of inclusion and exclusion of articles [9].

To ensure that all relevant articles were included, the research team re-examined the titles of the original set of 493 publications identified in the previous study. This led to the identification of two additional eligible articles, bringing the total number of articles for this study to 38. During initial screening, three articles were identified as study protocols [14,15,16] that did not report outcomes, so these were excluded from further analysis. In addition, two articles did not meet the inclusion criteria and were excluded, one reported development of a resource but did not implement the resource in practice [17], while the other study did not aim to improve Indigenous health outcomes [18]. Table 1 summarises the inclusion and exclusion criteria for this narrative review. Figure 1 summarises the process of inclusion of papers in this review.

### 2.2. Data Extraction

Authors SB, KPF, AM, EW, and SCT reviewed the papers. We extracted information on the nature of the interventions, key concepts that worked and did not work in the studies, and reflections on the intervention processes using a data extraction table created in Microsoft Word for Microsoft 365 MSO (Version 2508). This information was then cross-checked by at least one additional author to ensure accuracies. Any discrepancies were resolved through discussion within the wider group. Information about the study design, geographic remoteness of the research, and the state where the research was conducted were also extracted.

### 2.3. Data Analysis

A thematic analysis was conducted to identify key themes and learnings from the intervention studies. Thematic analysis followed Braun and Clarke’s six phase framework [19], described in Table 2.

## 3. Results

### 3.1. Characteristics of the Papers Reviewed

Thirty-three studies were included in this review. The intervention studies showed considerable heterogeneity. The papers described four types of interventions: novel health interventions (*n* = 9) [22,23,24,25,26,27,28,29,30], improvements to existing interventions (*n* = 5) [31,32,33,34,35], improvements to existing systems (*n* = 6) [36,37,38,39,40,41], and workforce interventions (*n* = 13) [42,43,44,45,46,47,48,49,50,51,52,53,54]. Supplementary File S1 displays a list of the 33 intervention studies included in this review. Studies were conducted across multiple levels of remoteness and across various states in Australia, with a large proportion located in Western Australia (*n* = 11, 33%) (Table 3). Most studies (*n* = 30, 91%) were co-authored by UDRH authors in partnership with university colleagues and industry collaborators; however, the specific role of UDRH staff in delivering or leading the interventions was not always clearly described. Only three studies were authored entirely by UDRH staff. The study designs ranged widely and included qualitative evaluations, mixed-method designs, cross sectional, observational, case studies, longitudinal trials, quasi-experimental, and randomised controlled trials (RCTs). Supplementary File S1 outlines the study design for each article. Read and colleagues paper [34] described the same RCT as Ralph et al. [33]. The duration of interventions ranged widely, from single session interventions to ongoing interventions that were embedded into systems. Details of intervention length are provided in Supplementary File S1.

### 3.2. Key Themes and Learnings

Supplementary File S1 summarises the nature of the interventions and key learnings extracted by authors. These concepts were synthesised into four themes: principles of engagement and design, considerations for improving healthcare systems, considerations for improving healthcare workforce, and the sustainability of interventions and improvements in outcomes. A summary of these themes is presented in Table 4.

#### 3.2.1. Principles of Engagement and Design

Effective engagement and design principles are crucial for the success of interventions in Indigenous health. Key concepts included building and strengthening partnerships and collaboration [23,25,27,30,31,33,34,36,37,39,40,42,43,54], implementing multi-component interventions [26,28,32,34,46,47,54], effective planning of resources and processes [27,42,50,51,52], awareness of cultural sensitivities [25], and adapting content and delivery to be culturally and literacy level appropriate [25,27,29,30,42,43,45,47,54].

##### Building and Strengthening Partnerships

Many studies illustrated the importance of partnerships and collaborations between Indigenous communities and stakeholders in designing and implementing interventions. Twenty-two studies described partnerships with Indigenous people [22,23,25,27,29,30,31,35,36,37,39,40,42,43,44,45,46,48,50,51,52,54]; however, the quality or involvement of these partnerships and co-design methods were often not detailed. Three studies described participatory research methods [23,43,46]. A few studies demonstrated long standing partnerships with Indigenous communities: Rae and colleagues (more than nine years) [30], Durey and colleagues (more than eight years) [37], Isaacs and Lampitt (five years) [27]. Kong and colleagues [46] reported a co-design process over a period of three years. Some studies [25,31,54] reported poor engagement when interventions were not aligned with the priorities of Indigenous communities.

##### Multi-Component Interventions

Studies [26,28,32,34,46,47,54] indicated that multi-component interventions are needed, as issues are usually complex and multi-faceted. Interventions need to consider both systemic and individual factors of healthcare workers and patients to be most effective.

##### Preparation, Planning and Resourcing

It is essential that those delivering interventions are adequately trained and that confidentiality is addressed, particularly in small communities where patients and healthcare workers interact with each other outside of healthcare contexts [25]. It is also important that interventions are well-planned and resourced, with checklists for facilitators to ensure adherence to protocols [42], that participants have adequate access to resources such as electronic devices required to participate in the intervention [42], that those delivering the intervention are experienced at managing conflict, particularly when there are sensitive topics [50,51], are adequately resourced to follow-up those identified as requiring care [27], and that the resources and materials used are appropriate for the literacy levels of participants.

#### 3.2.2. Considerations for Improving Healthcare Systems

Key learnings about improving healthcare systems included increasing efficiency of healthcare services [35,38,41], reducing barriers to attendance [24,26,42,54], using interpreters to improve engagement and outcomes [32], fostering top-down organisational cultural security [42], enhancing development of cultural security in systems and developing staff understanding and empathy for cultural determinants to improve the engagement of Indigenous people [23,24,25,27,29,30,32,33,42,43,45,47,54].

##### Increasing Efficiency of Healthcare Services

Efforts to increase the efficiency of healthcare services can improve engagement in healthcare for Indigenous people. One study, which integrated health promotion, assessments and chronic disease management in primary healthcare services, was successful in increasing the engagement of Indigenous people, improving the quality of care, and reducing mortality [39]. Three studies used point-of-care testing to increase efficiency by providing test results within the timespan of the patient’s appointment; all three studies found positive impacts, in treating sexually transmitted diseases [38], managing diabetes [40], and reducing the risk of stroke in rheumatic heart disease patients [41]. Integrating effective use of technology in addition to multicomponent care increased the efficiency of appointments. A study implemented an ear health pathway for children with protocols for integrated pathways of care covering, referrals, nurse care and education, and ear specialist reviews in one program; this increased engagement and reduced waiting list times including through effective use of telehealth systems and software [35].

##### Reducing Barriers to Attendance

When repeated appointments are required, reducing barriers to attendance such as accessibility to healthcare services increased engagement. Flexible models of service in remote communities and providing transportation reduced barriers to attendance and increased engagement. It was noted that transport by external services such as taxis was unreliable, and there was a need to cater to mobility impaired individuals [24]. Providing outreach services, in which specialist services are brought into remote communities, increased engagement and outcomes in dental care in school-aged children [26].

##### Interpreters

Interpreters are valuable assets in improving inclusivity, as they can help patients communicate, understand, and engage with healthcare workers and therefore improve adherence to treatment resulting in better outcomes. It is important that interpreters are part of teams and that clinicians are educated about referral pathways and benefits of use of interpreter services [32].

##### Organisational Leadership and Cultural Security

Organisational leadership was an important factor in improving cultural security, as leaders can support initiatives by providing resources and pathways for implementation and reducing barriers to implementation [42]. Engaging staff to attend non-mandatory training sessions was challenging, particularly when those encouraging the attendance were not in leadership roles [32]. One study demonstrated a systems-based approach driven by organisational leaders and were successful in improved Indigenous healthcare in community and hospital settings [37]. Another study reported that participants requested that interventions be delivered by Indigenous health services, due to the paucity of staff understanding about cultural needs [25]. This highlighted the importance of developing staff understanding and empathy for cultural determinants. One study demonstrated that staff education about appropriate ways of engaging and communicating was essential in developing culturally responsive services [23]. Conducting audits and clinical quality improvement exercises was also beneficial to improving cultural security [36].

#### 3.2.3. Considerations for Improving Healthcare Workforce

Considerations for improving the healthcare workforce included appropriate mentorship and support [44], ongoing capacity building [25,28,35,37,42,46,47,49], experiential learning for healthcare workers/students [48,50,51,52,53], and the need for education about cultural safety and topics in Indigenous culture relating to healthcare [52].

##### Developing the Healthcare Workforce

Improving the healthcare workforce through staff education, retention measures, and ensuring cultural security in healthcare environments are important factors [33]. Recruitment of staff who were willing to work flexibly and reflexively to individual needs underpinned providing culturally safe care [24]. Organisations should have representation of both male and female staff [24] and ensure that staff are adequately qualified and trained to deliver culturally safe care [24,25,27,40,54].

Mentorship was identified by multiple studies as a crucial component in the capacity building of healthcare staff. A mentorship and support program for Indigenous nurses and midwives increased retention [44]. Mentorship of Indigenous interpreters was valuable in engaging multidisciplinary teams and patients [32], and mentorship was also important for non-Indigenous staff and students to develop culturally responsive services [23].

##### Ongoing Capacity Building

Studies highlighted the need for and significance of capacity building of Indigenous staff [35,44]. However, one study identified organisational barriers to Indigenous staff engaging with professional development opportunities, including prejudiced assumptions of managers [54]. Ongoing capacity building such as opportunities for skills and knowledge acquisition are vital. Capacity building contributed to increased job satisfaction and career progression in Indigenous staff [44] and increased confidence in providing healthcare [46]. Lalloo and colleagues reflected that the capacity building of local staff would reduce the reliance on flying in external services to rural communities [28]. It is important that courses and training modules incorporate Indigenous knowledge through consultation with communities, to ensure that the content delivered is consistent with the social and cultural needs of Indigenous peoples [43]. Follow-up training sessions may be helpful to reinforce learnings [42].

##### Experiential Learning

Four studies demonstrated the value in educating healthcare students on Indigenous culture and determinants of health and highlighted the importance of experiential learning [50,51,52,53]. When students had the opportunity to interact with Indigenous people, pre-existing stereotypes were challenged in a way that was different to watching excerpts on film [50]. Smaller class sizes were more effective for change compared to large lecture theatres that usually gave students anonymity and opportunity for less participation [51]. Students also learnt relevant clinical and communication skills through experiential learning, developing an understanding that is difficult to gain in classroom settings [53]. Students indicated the need for additional education on applying cultural knowledge to their specific area of practice [50].

#### 3.2.4. Sustainability

Access to funding and limited resources appeared to be a common barrier to implementation and sustainability of the interventions [23,29,36,44].

Although some studies showed improvement in outcomes, the sustainability of improvements in the long-term were mostly unknown. The follow-up measurement periods ranged from immediately after the completion of the intervention to two years after commencement; however, many studies did not specify their follow-up period. Only one study analysed data over a long period of time (nine years) since commencement of the intervention and showed the value of audits and the use of clinical quality improvement tools to improve the quality of care [36].

## 4. Discussion

This paper reviews the key learnings from twelve years of Australian Indigenous health intervention research conducted by UDRHs. There was considerable diversity in the work, with focus on improving health outcomes for Indigenous communities and the capacity building of Indigenous people and the health workforce. As well as a local focus, there were efforts at creating system changes. Most of the studies were qualitative in nature, and many implemented mixed-methods designs. Key learnings about principles of engagement and design, considerations for improving healthcare systems, considerations for improving healthcare workforce, and the sustainability of interventions and improvements were derived from the articles.

Interventions were more likely to be successful when they targeted the underlying biological, psychological, social, environmental, and economic determinants that align with priorities of Indigenous health. It was no surprise that some studies had poor participation in interventions when they were not aligned with the priorities of Indigenous communities [25,31,54]. Inclusion of Indigenous communities and Indigenous workforce during design, implementation and evaluation stages are an integral part of ethical and culturally secure practice [55,56]. Genuine partnerships can take years to develop [23]; however, these partnerships are beneficial for all stakeholders involved. Indigenous communities benefit from having culturally secure interventions that meet community needs and priorities, while healthcare services can overcome barriers to engagement and provide a high quality of care to Indigenous people. The long periods of partnerships with Indigenous communities seen in some studies [30,37,46] are consistent with the decades of UDRH partnerships with local Indigenous communities [57]. Due to the time-intensive nature of developing genuine partnerships, resources need to be allocated for the co-design process [23]. The Australian Government in collaboration with Indigenous stakeholders have published a National Aboriginal and Torres Strait Islander Health Plan [10], indicating areas of priority for healthcare; this framework will be important for informing future interventions.

Interventions that addressed underlying determinants were successful in increasing the engagement of Indigenous people; for example, one study identified that providing patient transportation was an important component of the intervention, as it addressed the barrier of access to the service [24]. Increasing the efficiency of healthcare appointments also increases the accessibility of care in regional and remote communities [35,38,41]. Healthcare services noted that Indigenous patients often fail to attend or are lost to follow-up [58]. Therefore, it is important to reduce the number of appointments that patients are required to attend and adopt processes that reduce barriers to attendance, especially for conditions where multiple appointments are required. While such service alignment is pertinent to the general population, the constellation of barriers for Indigenous people often relates to underlying determinants and health service engagement for health improvements and therefore requires a multi-faceted approach. Health interventions may consider addressing underlying determinants relating to poverty such as transport, overcrowding, low income, and poor hygiene [24], in combination with the targeted health-related issues. It is important to engage and educate caregivers when treating children, as caregivers have a key role in ensuring compliance and optimal care in children [35]. Literacy levels should be considered, as participants reported feeling shame about their inability to read or understand the materials [27]; eliciting feelings of shame is likely to be counterproductive. Interpreters are valuable in addressing communication barriers and can improve Indigenous patient engagement [32]. Patients with linguistically diverse needs can be helped by people who understand their languages and cultural needs, to advocate and propose appropriate changes in services and systems; the preference of Indigenous Australians for holistic approaches has long been described [59].

For interventions to be most effective, multiple components must complement each other to address multi-faceted issues [26,28,32,34,46,47,54]. O’Connor and colleagues demonstrated a good example of a multicomponent intervention implemented across individual and systemic levels [32]. The introduction of an Aboriginal Interpreter Coordinator and employment of interpreters were interventions implemented at a systemic level, whilst mentoring and support for interpreters and provision of education for healthcare workers about interpreter use were interventions for individuals that had a positive effect on patients [32]. The individual components of this intervention were unlikely to be as effective in improving outcomes in isolation.

When designing interventions, it is essential to consider which factors are enablers or barriers to change. For example, one study showed that price discounts on healthier foods resulted in increased purchase of healthier foods, while education in addition to the price discounts did not result in significant differences in behavioural change to discounts alone [22]. The study demonstrated that barriers to healthy behaviours were associated with being able to afford the items, rather than a lack of knowledge about healthy foods. It is not unusual that some families cannot afford nutritious foods due to limited finances [60], highlighting the importance of multi-component interventions to target underlying determinants that encompass individuals, systems and environments.

Researchers and clinicians should consider their duty of care responsibilities when implementing interventions. For example, while one study reported culturally welcoming and congruent engagement, there was no apparent process for referral or follow-up of distressed individuals [27]. Appropriate resources must be allocated for follow-up pathways to adequate treatment and support for vulnerable individuals. When studies aim to detect a health issue but do not treat the issue, those identified as requiring further assistance must be provided with pathways to seek professional care.

Organisational leadership plays a key role in promoting culturally secure healthcare and enabling the sustainability of interventions. Studies noted difficulties with implementing interventions due to limited resources and organisational support [23,29,32,36,44]. Interventions need to be embedded on a systemic level, as external funding and resources may be temporary. One study noted that when funding for the project had stopped, there was no facilitation and support; so, the use of the program and tools reduced [36]. Another study identified that prohibitive policies and a lack of fit with workplace context, culture and philosophy were barriers to implementing the intervention and acknowledged the importance of receiving support from organisational leaders [42]. The delivery of the interventions should not be person-dependent; for example, one study noticed a reduction in referrals and sessions when their nurse was on extended leave [35]. The wider research literature has shown that organisations that are successful at service integration have leaders who champion and support joint goals and shared vision [61,62]. Leaders can shape the organisation functionally and structurally to adapt to new requirements that result in improvements [62], and formal change management processes that include logistics, resources and funding models are key to successful service integration [61].

In addition to caring for patients, it is in the best interest of organisations to build the capacity of their employees, through staff education, recruitment, retention measures, and ensuring cultural security [24,25,27,33,40,54]. Improving Indigenous healthcare must involve the capacity building of Indigenous and non-Indigenous staff [23,35,44] and address barriers for Indigenous staff, such as racism and prejudice of managers [54]. Capacity building is an ongoing process in which people and organisations increase their abilities to perform core functions, solve problems, define and achieve objectives, build networks and knowledge, and understand and address their development needs in a sustainable manner [63]. Continuing professional development (CPD) is an important element of capacity building through updating healthcare workers’ knowledge and skills and is related to providing high quality evidence based care [64]. CPD can be formal or informal and can be mixed and multifaceted, ranging from training and coaching to mentoring [64]. Mentorship is an important element of capacity building; studies showed that mentoring improved the retention of Indigenous staff [44], was valuable in engaging multidisciplinary teams and patients to use Indigenous interpreters [32], and helped non-Indigenous staff and students to develop culturally responsive services [23]. Healthcare workers’ development of competencies is related to organisational learning cultures, in particular whether the organisation provides an active learning climate in which workers feel psychologically safe and are stimulated to ask questions, seek feedback, experiment, and reflect on results [65]. Organisational leaders should foster a positive environment for learning, by ensuring allocated time, creating a culture of supporting each other in daily tasks, and having ample opportunities for learning [64].

As students build the future of the professional workforce, they are likely to have influence on provision and improvement to Indigenous health as they progress through their career in healthcare organisations. Educating students to become culturally competent university graduates can improve Indigenous health and reduce socio-economic disparities [66], as culturally safe environments improve the engagement of Indigenous people. Studies showed the potential for early intervention through education about Indigenous culture and healthcare during university courses, as workshops were effective at challenging racism and engaging non-Indigenous students in difficult discussions that encourage them to become allies of Indigenous people and created opportunities for students to become mindful of cultural differences in clinical practice [50,51,52,53]. A single first-year unit about Indigenous culture was insufficient, as many students reported poor confidence in caring for Indigenous patients years after completing the first-year unit and reported gaps in their knowledge about Indigenous practices specific to their area of healthcare [52]. Experiential learning opportunities were particularly important, as students that completed placements on country reported learning valuable skills and lessons that were unable to be taught in a classroom [53]. Studies in this review highlight the importance of teaching and challenging beliefs of about Indigenous cultures and practices in small groups, through personal experiences, and engagement with Indigenous people, rather than through lectures or online materials that can leave participants feeling detached and impassive.

### 4.1. Limitations

A collaborative and capacity building approach underpinned this research. While this inclusive methodology strengthened the study, it extended the timeframe for analysis and manuscript preparation. As a result, additional relevant UDRH publications have undoubtedly been published since the initial article selection and completion of the analysis that are therefore not captured in this review.

While all papers in this review included at least one author affiliated with a UDRH, it was often unclear how the UDRHs were involved in the implementation of the interventions described. Similarly, although many studies referred to partnerships with Indigenous communities and researchers, limited detail was provided about the nature and depth of those partnerships—making it difficult to determine whether engagement involved consultation only or extended to co-design and community leadership. The key learnings derived may be biased according to the authors’ interpretations, as information about processes and outcomes were vague in many papers, particularly if studies were part of larger projects and were ongoing.

Some interventions showed positive effects on improving Indigenous healthcare; however, it is unclear whether the interventions were effective in achieving sustainable improvements over the long-term (i.e., years), as there was a lack of studies with long-term follow-up periods. Future studies should consider planning for long-term follow-up periods to evaluate the effectiveness of interventions and to establish sustainable improvements in Indigenous health.

The study designs were mostly qualitative in nature. Qualitative studies provide participant perspectives and interpretations, which give authors insight to participant experiences of the interventions. Whilst this rich information is valuable to reflect successes and identifying areas needing improvements, qualitative studies are unable to objectively measure the impact of the interventions (e.g., through effect sizes). Future studies should consider culturally appropriate measurement tools and study designs to measure the impact of interventions and allow for comparisons between different interventions. RCTs are generally considered to be the ‘gold standard’ of study designs; however, RCTs may not be the most suitable study design for Indigenous populations due to complex social and cultural factors [67]. In order to be sensitive to the complex social and cultural determinants experienced by Indigenous Australians, future studies should utilise more recently developed quality tools (e.g., The Aboriginal and Torres Strait Islander Quality Appraisal Tool) [21] to guide best practice in their prospective design and implementation of interventions in Indigenous health.

### 4.2. Recommendations

A number of recommendations can be made based on the key learnings from the body of UDRH intervention research (Table 5).

## 5. Conclusions

Over the past twelve years, UDRHs have made substantial and varied contributions to Australian Indigenous health intervention research. Their work has spanned a wide range of areas, including patient and community-focused interventions, workforce development, and health systems improvements. Their efforts have focused on improving health outcomes for Indigenous communities, the capacity building of Indigenous people and the broader health workforce, and aligning with the objectives of the Closing the Gap initiative [68], to improve Indigenous health.

This paper has provided a number of recommendations based on key learnings from the body of UDRH intervention research. Improving Indigenous health outcomes requires interventions grounded in meaningful community engagement and co-design, delivered through culturally safe practice and supported by a well-prepared workforce. Approaches should be multicomponent, addressing social determinants of health and reducing structural barriers to access. The long-term impact depends on adequate resourcing, strong organisation leadership and the integration of programs within healthcare systems to support the continuation of care and sustained capacity building. By implementing these recommendations, interventions can better address disparities and contribute to meaningful improvements in Australian Indigenous health. While these principles can be applied generally, UDRHs are in a unique position to advocate and influence the development of relevant and effective interventions for Indigenous people, through their roles in training the next generation of health professionals including Indigenous people and their partnerships with existing health services. UDRHs should reflect on their current and future projects to ensure that principles of engagement and design, considerations for improving healthcare systems, considerations for improving healthcare workforce, and the sustainability of interventions and improvements in outcomes are accounted for.

## Figures and Tables

**Table 1:** Inclusion and exclusion criteria.

	Included	Excluded
Time Period	2010–2021	Published prior to 2010 and after 2021
Language	English	Languages other than English
Type of article	Peer-reviewed full text articles describing intervention outcomes	Grey literature, conference procedures, published abstracts only, study protocols, commentaries
Scope	Intervention studies that aim to change an outcome in Indigenous health, including clinical interventions, community interventions and health promotion, systemic interventions, service delivery interventions	Non-intervention studies, such as studies that measure/describe problems or symptoms only without any actions aimed to change an outcome Interventions that do not consider Indigenous health in the study design
Article authorship	At least one author was affiliated with a UDRH	No authors have UDRH affiliation
Setting/location	Australia	Outside of Australia

**Table 2:** Thematic analysis process using Braun and Clarke’s framework [19].

Phase	Actions Taken by the Research Team
1: Familiarising with the data	Authors read and re-read the papers and noted down initial ideas about the papers.
2: Generating initial codes	Authors extracted excerpts from each paper into a table. Authors answered the following questions for each paper in a table: What was the nature of the intervention? Why did they do the intervention? What worked? What did not work? What recommendations were made?
3: Searching for themes	Authors summarised the information in phase 2, answering the following question for each paper: What were the key learnings from this paper?
4: Reviewing themes	Similar key concepts were grouped together, and authors identified over-arching themes of the key concepts.
5: Defining and naming themes	The themes were named to summarise the key concepts that were grouped together. Authors discussed these, referenced the excerpts from the papers when in doubt and came to a consensus.
6: Writing the report	Informed by existing frameworks [10,20,21] and following team discussions, the authors discuss the findings in this paper.

**Table 3:** Summary of study locations.

Location Remoteness	Number of Publications
Multiple levels of remoteness	9
Remote or very remote	8
Inner or outer regional	6
Major city	5
Unclear	5
**Location by state**	
National (Australia wide)	1
Research conducted across multiple states	4
New South Wales (NSW)	6
Northern Territory (NT)	6
Queensland	2
Victoria	2
Western Australia (WA)	11
Unclear	1

**Table 4:** Themes and key concepts identified.

Themes	Key Concepts
Principles of engagement and design	Importance of building/strengthening partnerships and collaboration Follow-up is needed to investigate long-term effects Multi-component interventions are needed Importance of well-planned interventions Importance of adaptations of content/delivery to be appropriate for target groups
Considerations for improving healthcare systems	Approaches to increase efficiency of healthcare appointments When repeated appointments are required, reducing barriers to attendance are important in increasing engagement Improved engagement of Indigenous patients is enhanced by developing cultural security in systems and educating staff/students Top-down organisational cultural security is essential Use of interpreters is valuable
Considerations for improving healthcare workforce	Importance of ongoing capacity building Importance of mentorship and support for the current Indigenous workforce Importance of experiential learning Need for inclusion of detailed/specific education about topics in Indigenous culture
Sustainability	Sustainability of interventions Sustainability of improvements in outcomes

**Table 5:** Recommendations.

Themes	Recommendations
Principles of engagement and design	Ensure meaningful engagement with Indigenous communities so that interventions align with local needs, priorities, and cultural values. Incorporate co-design, shared decision-making, and community leadership. Include Indigenous stakeholders in the design and implementation of interventions. Address structural barriers such as access to services, availability of resources, and varying literacy levels to support equitable participation. Design multicomponent interventions that combine system-level change with individual-level supports to address complex health challenges. Target underlying determinants of health—including economic, social, psychological, and environmental factors—to enhance participation and long-term effectiveness.
Considerations for improving healthcare systems	Embed interventions within existing healthcare systems to promote integration and continuity of care. Ensure adequate and sustained resourcing to support implementation and scale-up. Establish clear pathways for referrals and ongoing care to maximise impact. Strengthen organisational leadership and cross-sector collaboration to support system-level change and capacity building. Leverage UDRHs’ partnerships with health services to advocate for and influence the development of relevant and effective Indigenous health interventions.
Considerations for improving healthcare workforce	Provide cultural safety training and experiential learning opportunities for leaders, staff, and students. Support workforce development initiatives that build capacity in Indigenous health intervention research and practice. Utilise UDRHs’ role in training the next generation of health professionals—including Indigenous health professionals—to strengthen culturally responsive practice.
Sustainability of interventions	Increase the likelihood of sustainability from the outset through adequate resourcing, systemic embedding of programs, ongoing funding models and clear pathways for ongoing care. Incorporate long-term follow-up periods to evaluate the effectiveness of interventions, and to establish sustainable improvements.

## Data Availability

No new data were created or analysed in this study.

## Supplementary Materials

The following supporting information can be downloaded at https://www.mdpi.com/article/10.3390/healthcare14050595/s1, Supplementary File S1: Summary of intervention research in Australian Indigenous health by UDRHs.
