# Supplementary material for: Contributions of Australian University Departments of Rural Health to Indigenous Health Intervention Research: A Narrative Review
Source: Healthcare (Basel). 2026 Feb 27;14(5):595. doi: 10.3390/healthcare14050595 (PMC12984620; doi:10.3390/healthcare14050595)
Supplement: Supplementary file 1 [file healthcare-14-00595-s001.zip › Supplementary File 1.pdf]

Contributions of Australian University Departments of Rural Health to Indigenous health intervention research: A narrative review

Supplementary File 1. Summary of intervention research in Australian Indigenous health by UDRHs

**Author (year) Location**	**Method and Study design**	**Study population**	**Brief description of the intervention**	**Duration of intervention & measurement period**	**Key learnings**
Bailie et al. (2017)[1] Multiple states	Mixed methods<br>Case study based on audit data, documentary evidence and stakeholder insights	175 primary healthcare services	ABCD program to enable the use of CQI tools to improve best practices and quality of care.	**Duration:** 9 years of data across 5 Australian states/territories<br><br>**Measurement period:** 2005-2014	**Principles of engagement and design:**<br>-none-<br><br>**Considerations for improving healthcare systems:**<br>• Clinical Quality Improvement exercises are important to identify strengths and gaps in healthcare systems which in turn can enable targeted of policy and procedure<br><br>**Considerations for improving healthcare workforce:**<br>• Healthcare workforce capacity building through providing evidence-based best-practice models.<br><br>**Sustainability of the intervention and outcomes:**<br>• Providing adequate resourcing for collaboration is important to ensure continuation of the intervention implementation.<br>• Funding was associated with uptake of the intervention: when funding stopped, the use of the program and tools also reduced.
Bennett-Levy et al. (2017)[2] NSW	Qualitative Interviews and reports on consultation sessions	21 Indigenous and 5 non-Indigenous health professionals	Staff education on strategies related to overcoming the barriers and strengthening the enablers to the adoption of e-mental health services.	**Duration:**<br>2- or 3-day training program with up to 5 consultation sessions<br><br>**Measurement period:**<br>At the end of the 6-month consultation period the session reports were collated in date order for each of the 7 consultation groups	**Principles of engagement and design:**<br>• Incorporating culturally appropriate training and support, collaboration of Indigenous community in service design and implementation, integration of traditional healing with e-mental health intervention, and concerns related to cultural competence and access to technology.<br>• Ensure that barriers to implementation are reduced, e.g., providing devices for the e-mental health service<br><br>**Considerations for improving healthcare systems:**<br>• The importance of organisational leadership in supporting the initiative, providing resources and pathways for implementation.<br>• Importance of effective planning and preparation of interventions, and include checklists for implementation.<br>• Importance of tailored resources for culturally and organisationally relevant content and resources.<br><br>**Considerations for improving healthcare workforce:**<br>• Capacity building and equipping staff with skills to overcome barriers and strengthening enablers to adopting e-mental health services.<br>• Provide post-training consulting support sessions to reinforce knowledge and skills.

Author (year) Location	Method and Study design	Study population	Brief description of the intervention	Duration of intervention & measurement period	Key learnings
					**Sustainability of the intervention and outcomes:**<br>• Sustainability will be determined by how well barriers are addressed, and enablers-which facilitate the integration of e-mental health services within Aboriginal and Torres Strait Islander health practices-are supported.<br>• Successful implementation is associated with increased access to mental health services, translating into improved health outcomes for Aboriginal and Torres Strait Islander communities.
Bennett-Levy et al. (2021)[3] NSW	Qualitative Community-based participatory research based on meeting notes, written feedback and interviews	Indigenous community leaders and Indigenous health workers	Digital social and emotional wellbeing: strategies that address cultural, social, and emotional needs of Indigenous populations; workshops for healthcare professionals about Indigenous mental health resources	**Duration:** Online resources, since 2015<br><br>**Measurement period:** feedback received in 2015 from staff led to further development; 2020 launched website but no formal evaluation evident.	**Principles of engagement and design:**<br>• Streamlined community consultation process, allowed for community-based participation using community infrastructure for input, and a phased engagement strategy.<br><br>**Considerations for improving healthcare systems:**<br>• Government funding influences the areas in which resources and services are developed.<br><br>**Considerations for improving healthcare workforce:**<br>• Integrating Indigenous knowledge and community engagement into healthcare training is vital for addressing the mental health needs of Indigenous communities and delivering culturally sensitive services to diverse populations.<br><br>**Sustainability of the intervention and outcomes:**<br>• The sustainability of the intervention is improved by incorporating insights from Indigenous communities, making sure that digital mental health initiatives are culturally relevant and backed by the communities they aim to support.
Biles et al. (2021)[4] NSW	Qualitative Interviews	Participants enrolled in a cultural mentorship program aimed at assisting Indigenous nurses and midwives	A mentoring program for Aboriginal and Torres Strait Islander nurses and midwives in a rural health district.	**Duration:** 12 months<br><br>**Measurement period:** At the beginning and end of the program	**Principles of engagement and design:**<br>• Consider barriers such as geographic remoteness, shift work, and additional workload of mentors.<br><br>**Considerations for improving healthcare systems:**<br>• Mentorship programs can build Indigenous staff confidence to speak up about issues relating to culturally safe practices in health administration and leadership.<br><br>**Considerations for improving healthcare workforce:**<br>• Capacity building and support for existing Indigenous staff can help increase job satisfaction and career progression.<br>• Frameworks and funding need to be adequately supported for the success of mentorship programs.<br><br>**Sustainability of the intervention and outcomes:**

Author (year) Location	Method and Study design	Study population	Brief description of the intervention	Duration of intervention & measurement period	Key learnings
					• The program led to a higher retention rate of Aboriginal and Torres Strait Islander nurses and midwives, showing that targeted support and culturally specific mentoring can foster trust and create safe work environments.
Brimblecombe et al. (2017)[5] NT	Quantitative Stepped-wedge randomised controlled trial	20 remote community stores	A 20% price discount on food and drink with and without consumer education, in a socioeconomically disadvantaged population.	**Duration:** 24 weeks for the intervention in step-wedge approach<br><br>**Measurement period:** July 1st 2012 – Dec 28th 2014<br>49-week baseline data-collection phase, followed by 24-week intervention phase and a 24-week post intervention follow-up. Intervention was rolled out in five groups of four stores at 8 week intervals.	**Principles of engagement and design:**<br>• More creative approaches to promoting fruit and vegetables in-store are needed, such as retailer-led merchandising for health together with programs to build consumer confidence to purchase more vegetables and discouraging purchase of less healthy foods.<br>• Other food-related interventions are needed, including cooking and food budgeting programs, and improved household food preparation and storage infrastructure.<br><br>**Considerations for improving healthcare systems:**<br>• Price discounts improved purchases of fruit and vegetables and bottled water, but not diet soft-drinks.<br>• Consumer education in addition to discounts did not make significant differences in consumer behaviours overall; discounts alone appeared to be the most influential mechanism of change.<br><br>**Considerations for improving healthcare workforce:**<br>-none-<br><br>**Sustainability of the intervention and outcomes:**<br>• Price discounts increased purchase of fruit and vegetables, and behaviour persisted after removal of the discount (but not clear for how long)
Cairns et al. (2022)[6] Qld	Mixed methods Action research	Two remote communities	A locally based community rehabilitation and lifestyle service.	**Duration:** 2 years overall for two cycles of planning for and trialling the initial service and collecting feedback, followed by replanning and implementing the updated service (unspecified engagement time)<br><br>**Measurement period:** Service was initially trialled for 6 months and the planning in implementation of the first cycle was the reported.	**Principles of engagement and design:**<br>• Recognising the risk of developing a service that would fit a western world view of health service delivery, changes were made to align the research with an Aboriginal Research Framework. An action-research approach to co-design led to the establishment of a unique community rehabilitation service to address disability and rehabilitation needs.<br>• Co-design takes a long time: it took 2 years (longer than most project or research funding allowed), therefore in-kind funding was needed to initiate and progress this genuine codesigned service.<br><br>**Considerations for improving healthcare systems:**<br>• Culturally responsive community rehabilitation services in Aboriginal and Torres Strait Islander communities requires holistic and community-wide perspectives of wellbeing.

Author (year) Location	Method and Study design	Study population	Brief description of the intervention	Duration of intervention & measurement period	Key learnings
					**Considerations for improving healthcare workforce:**<br>• Education about Aboriginal and Torres Strait Islander ways of engaging and communicating, and leadership and mentorship for non-Indigenous allied health professionals and students are essential components to develop culturally responsive services.<br>• The service was changed in response to findings of the evaluation: employment of a dedicated allied health rehabilitation supervisor and local Indigenous community rehabilitation co-worker.<br><br>**Sustainability of the intervention and outcomes:**<br>• Funding was required for the positions, plus a vehicle, mobile phones and therapy consumables – likely to be needed for ongoing sustainability of the intervention.
Carey et al. (2016)[7] NT	Qualitative Semi-structured interviews	20 participants – 5 patients and 5 carers, 7 referrers and 3 stakeholders	A community based, culturally appropriate palliative care respite service. Patients were able to access the service when they needed to rather than attending a prescribed program and staff spent time understanding the individual requirements, including the cultural considerations of each patient.	**Duration:** 10 months<br><br>**Measurement period:** 10 months after the service commenced	**Principles of engagement and design:**<br>• Important principles of practice such as flexibility and cultural appropriateness to increase engagement of Indigenous people.<br>• The facility was staffed by experienced health professionals who took the time to enquire about the perspectives of the individual patients and carers and they used these perspectives to inform their practice.<br>• Importance of staff retention/consistency: Many participants identified that trusted relationships that were built between patients and the consistent pool of respite staff were an integral part of successfully engaging people with the program.<br><br>**Considerations for improving healthcare systems:**<br>• A flexible, community based, culturally appropriate palliative care respite service can improve quality of life for respite patients and their carers.<br>• The respite service enabled improved care coordination of chronic and complex patients as well as improved medication compliance and symptom management.<br>• Providing reliable transport would assist patients in attending the service on a regular basis.<br>• Exploring options to cater for overnight attendances at the facility was seen as a desirable initiative for the future.<br>• Provision of regular support with medicines, wound care and other health care needs helped to stabilise people and prevent acute exacerbation of illness<br><br>**Considerations for improving healthcare workforce:**<br>• Willingness of the staff to work flexibly and responsively ensured that marginalised people were able to access the service in culturally appropriate ways. This was an important factor in the positive impact of the service.

Author (year) Location	Method and Study design	Study population	Brief description of the intervention	Duration of intervention & measurement period	Key learnings
					• Ensuring that appropriately trained staff are recruited and that a representation male and female staff is important.<br><br>**Sustainability of the intervention and outcomes:**<br>-none-
Chapple et al. (2016)[8] Multiple states	Qualitative Semi-structured interviews	10 Aboriginal volunteer clients	Living Well Smoke Free (LWSF) training – a smoking cessation program adapted for Aboriginal and Torres Strait Islander people	**Duration:** This was a once off pilot, participants received one condensed version of the session.<br><br>The intention was that there would be 6-8 sessions with a health worker.<br><br>**Measurement period:** One post-intervention interview of approximately 30 minutes each from 10 participants<br>Three participants were contacted by phone after the intervention (unspecified time) interviews were over a period of 6 months.	**Principles of engagement and design:**<br>• The majority of participants identified the importance of having support when changing behaviour, which was an important component of the intervention.<br>• Education is an important component of enacting change: Participants reported that having more knowledge about smoking and how it impacts on health made them more likely to quit smoking.<br>• The program was adapted with Aboriginal and Torres Strait Islander members of the National Advisory group, which resulted in participants reporting that content was culturally appropriate.<br>• Consider addressing selection bias of participants: those who volunteered were already interested in giving up smoking, and people with complex health and psychosocial difficulties did not view giving up smoking as a priority.<br>• Consider confidentiality issues.<br>• Consider addressing multiple factors that influence smoking cessation: length of time smoking, smoking as part of a lifestyle, having a partner that also smokes, managing emotions, finances, worry about withdrawal symptoms<br><br>**Considerations for improving healthcare systems:**<br>• Many of the participants advocated for the intervention to be delivered in an Indigenous health service where the workers have more empathy and understanding of their cultural needs.<br><br>**Considerations for improving healthcare workforce:**<br>• Provide adequate training for staff delivering the interventions.<br><br>**Sustainability of the intervention and outcomes:**<br>-none-
Durey et al. (2016)[9] WA	Qualitative Interviews and a yarning circle	60 participants – 30 Aboriginal Health Action Group members, 12 Aboriginal service users, 4 health providers of Aboriginal services, 14	District Aboriginal Health Action Groups collaborated with health service providers to design culturally responsive care	**Duration:** 12 months<br><br>**Measurement period:** in 2012	**Principles of engagement and design:**<br>• Having processes that are driven and owned by the community can be successful<br>• Ongoing funding should build on and strengthen existing partnerships.<br><br>**Considerations for improving healthcare systems:**<br>• Participants reported that health services improved in community and hospital settings, leading to increased access and trust in local health services.

Author (year) Location	Method and Study design	Study population	Brief description of the intervention	Duration of intervention & measurement period	Key learnings
		mainstream health service providers			• Indigenous people are included in decision making about health care and, where possible, their views had influenced improvements to health services.<br><br>**Considerations for improving healthcare workforce:**<br>• Employment of Indigenous people in the workforce<br>• Ongoing capacity building through training, support and mentoring is needed to build knowledge, skills and experience, and increase employment opportunities for Indigenous people.<br><br>**Sustainability of the intervention and outcomes:**<br>• Ongoing sustainability of the DAHAGs requires ongoing capacity building and mentoring and funding
Fernando et al. (2021)[10] Unclear	Quantitative Longitudinal trial, biomedical measures	208 Indigenous children	Dental intervention including: detailed dental examination, investigation of salivary biomarkers, provision of necessary restorative treatment, two annual applications of fissure sealant to appropriate teeth, swabbing teeth with povidone iodine, and application of fluoride varnish.	**Duration:** Two occasions, once annually<br><br>**Measurement period:** Two years	**Principles of engagement and design:**<br>• Consider interventions for addressing underlying social determinants for poor school/healthcare appointment attendance: children's attendance at school fluctuated due to social or cultural reasons, and truanting from schools is widespread, attendance rates of attending multiple dental appointments are poor.<br><br>**Considerations for improving healthcare systems:**<br>-none-<br><br>**Considerations for improving healthcare workforce:**<br>-none-<br><br>**Sustainability of the intervention and outcomes:**<br>• For effective and lasting impact, the challenges faced by these communities related to wider public health access and service provision in the context of relevant social and cultural factors need to be addressed with multidisciplinary interventions.
Guy et al. (2018)[11] Multiple states	Quantitative Randomised controlled trial	860 Indigenous patients aged 16-29 years who tested positive for Chlamydia trachomatis or Neisseria gonorrhoeae. Patients were	Point-of-care testing for chlamydia and gonorrhoea	**Duration:** 24 months (12 months usual care vs. 12 months intervention)<br><br>**Measurement period:** June 2013 – Feb 2016	**Principles of engagement and design:**<br>• Point of care testing for chlamydia and gonorrhoea in remote primary health services improves time to treatment<br><br>**Considerations for improving healthcare systems:**<br>• Point of care testing can improve the efficiency of health services and improve early detection of sexually transmitted diseases.<br><br>**Considerations for improving healthcare workforce:**

Author (year) Location	Method and Study design	Study population	Brief description of the intervention	Duration of intervention & measurement period	Key learnings
		from 12 health services.			-none-<br><br>**Sustainability of the intervention and outcomes:**<br>-none-
Haigh et al. (2016)[12] WA	Qualitative Interviews and focus group discussions	67 participants – 5 individuals involved in the development of the DVD, 17 health professionals, 45 Aboriginal community members	An educational DVD about bowel cancer screening	**Duration:**<br>One session<br><br>**Measurement period:**<br>Follow up interviews to assess views and impact of watching the resource (unspecified period)	**Principles of engagement and design:**<br>• There was limited engagement of Indigenous community members and health providers, as their focus was directed towards more pressing health care issues.<br><br>**Considerations for improving healthcare systems:**<br>• Interest in the resource might be increased once the Indigenous component of the screening program is more closely linked with primary care.<br><br>**Considerations for improving healthcare workforce:**<br>• Takes pressure off health workers because it is easier to just show a DVD rather than engage community members.<br><br>**Sustainability of the intervention and outcomes:**<br>• Easy to use, but no real buy in from providers or meaningful engagement with Indigenous people.
Isaacs & Lampitt (2014)[13] Vic	Mixed methods Kessler 10 questionnaire and observational	17 rural Aboriginal men	Koorie Men's Health Day: medical examination, blood test for diabetes and psychological assessment using the Kessler-10 questionnaire	**Duration:**<br>One session<br><br>**Measurement period:**<br>At time of intervention only	**Principles of engagement and design:**<br>• Importance of building on existing relationships with Indigenous communities: the lead researcher already had built trust with the local community for over 5 years.<br>• Partnerships between local Indigenous l community and non-Aboriginal stakeholders were important in conducting the intervention.<br>• Consider different levels of literacy levels of participants: Low literacy affected how the K10 was answered<br><br>**Considerations for improving healthcare systems:**<br>• When screening for mental health difficulties, ensure adequate resourcing to conduct follow ups for those requiring further support: This study was under-resourced to conduct follow-ups for individuals who needed further support.<br><br>**Considerations for improving healthcare workforce:**<br>• It was important that cultural safety training was provided to staff.<br><br>**Sustainability of the intervention and outcomes:**<br>-none-

Author (year) Location	Method and Study design	Study population	Brief description of the intervention	Duration of intervention & measurement period	Key learnings
Khalil (2019)[14] Vic	Mixed methods Before-after knowledge, confidence, behaviour, utilisation of the medication safety program and resources	17 Aboriginal Health Practitioners	Medication safety education tailored for Indigenous health practitioners	**Duration:**<br>Two steps: Stage 1 = interviews conducted between October 2016 and December 2016.<br><br>Stage 2 = A 2-day educational program and an online resource for AHP to access in the workplace.<br><br>**Measurement period:**<br>Completion of a questionnaire before and 6 months after the program implementation	**Principles of engagement and design:**<br>• Culturally acceptable materials for Indigenous staff<br><br>**Considerations for improving healthcare systems:**<br>• Education about medication safety decreased medication-related incidents.<br>• Importance of assessment systems with feedback mechanisms for improvements.<br><br>**Considerations for improving healthcare workforce:**<br>• Using a team approach, involving experienced educator who was familiar with Indigenous issues while being aware of staff workplace culture and expectations helped with staff engagement.<br><br>**Sustainability:**<br>• For long term outcomes, the program should be incorporated into current usual healthcare practices. Organisations need to provide ongoing training and support for AHPs, foster a culture of safety, establish continuous feedback mechanisms, and ensure the commitment of healthcare organisations and community stakeholders to prioritise medication safety in rural areas.
Kong et al. (2021)[15] NSW	Mixed methods, Community based participatory research. Pre-post Questionnaire.	7 Aboriginal Health Workers	"Grinnin' Up Mums & Bubs": train Indigenous Health Workers to promote oral health among Aboriginal and Torres Strait Islander pregnant women.	**Duration:** 12 months<br>3 training workshops delivered due to conflicting schedules of the AHW and were for 1 or two hours.<br><br>A pre-questionnaire was administered prior to the workshops and were verbally discussed between six and 20 minutes,<br><br>**Measurement period:**<br>At the beginning and at the end of the program	**Principles of engagement and design:**<br>• The content of the training was easy to understand, the length of the training was adequate, relevant to their work, and built on staffs' existing knowledge.<br>• Culturally relevant resources were developed by Indigenous health workers and a local Indigenous graphic designer.<br><br>**Considerations for improving healthcare systems:**<br>• Culturally safe practices, enhanced training for AHWs and strong community engagement are essential for health services to effectively meet the needs of Aboriginal and Torres Strait Islander women during pregnancy.<br><br>**Considerations for improving healthcare workforce:**<br>• Capacity building through increasing knowledge and confidence in Aboriginal staff in promoting oral health care, using culturally appropriate materials.<br>• The intervention effectively enhanced midwives' knowledge and confidence in promoting oral health, improved maternal oral health and dental service uptake during pregnancy, and was widely acceptable, feasible, and cost-effective for all stakeholders.<br><br>**Sustainability of the intervention and outcomes:**

Author (year) Location	Method and Study design	Study population	Brief description of the intervention	Duration of intervention & measurement period	Key learnings
					• Sustainability is enhanced by its integration into community health frameworks and the active participation of AHWs, promoting ongoing engagement and capacity building.
Lalloo et al. (2021)[16] Qld	Quantitative Non-randomised longitudinal trial	408 Indigenous children	Dental intervention: placement of fissure sealants on suitable teeth, and application of povidone-iodine and fluoride varnish to the whole dentition, following completion of any necessary restorative dental treatment. Standard diet and oral hygiene advice were provided. As above in Fernando et al., 2021	**Duration:** Once annually<br><br>**Measurement period:** Two years	**Principles of engagement and design:**<br>• Following extensive consultation with the community it was decided that a non-randomised trial would be most culturally appropriate.<br>• Importance of addressing other social determinants, to reduce the burden of poor oral health (e.g., consumption of sugar-sweetened beverages)<br><br>**Considerations for improving healthcare systems:**<br>• The dental intervention in this study significantly reduced advanced carious lesions in permanent dentition.<br>• Due to the resource intensiveness of the intervention, the paper suggested that fluoridation of water be reintroduced as a passive intervention.<br><br>**Considerations for improving healthcare workforce:**<br>• Local capacity building to reduce reliance on the services of fly-in dentists: The study suggested training community health workers to perform regular minimally-invasive interventions such application of povidone iodine applications and fluoride varnish.<br><br>**Sustainability of the intervention and outcomes:**<br>• Improvements are only likely to continue if dental treatment is continued to be delivered. Consider other passive interventions and addressing social determinants for improved long-term outcomes.
Lin et al. (2016)[17] WA	Mixed methods Pre/post cohort design with interviews and clinical audit	4 GPs at a rural Aboriginal Medical Service (AMS)	Education for General Practitioners for managing lower back pain (LBP): reducing unnecessary LBP radiological imaging referrals, enhancing psychosocial-oriented patient assessment, and increasing the provision of LBP self-management information to patients	**Duration:** 12 months<br><br>**Measurement period:** Over two six-month periods (pre- and during the intervention) July – Dec 2011 and July – Dec 2013	**Principles of engagement and design:**<br>• Importance of cultural sensitivity in developing trust and rapport between healthcare providers and Indigenous patients.<br><br>**Considerations for improving healthcare systems:**<br>• Consider integrating evidence-based tools into usual clinical systems (e.g., electronic clinical decision making tools integrated into usual care), to reduce barriers to implementation.<br><br>**Considerations for improving healthcare workforce:**<br>• Continuous education for healthcare providers, fostering collaboration between patients and providers, and developing community-based support systems were highlighted to improve patient outcomes and create a culturally sensitive approach to managing low back pain in Indigenous communities.

Author (year) Location	Method and Study design	Study population	Brief description of the intervention	Duration of intervention & measurement period	Key learnings
					• Enablers for change and barriers identified.<br><br>**Sustainability of the intervention and outcomes:**<br>• The integration of the intervention into existing health-care practice, continued support and training of the providers, community engagement, and resource availability.<br>• Greater awareness and familiarity of best practice among health providers were associated with better patient outcomes.
O'Connor et al. (2021)[18] NT	Mixed methods Audit of interpreter booking requests and hospital separations data, Pre-and post survey	127 hospital-based clinicians	Employment of an Aboriginal Interpreter Coordinator, 'Working with Interpreters' training for healthcare providers, and championing of interpreter use by doctors.	**Duration:**<br>12-month intervention<br><br>**Measurement period:**<br>4-year study (pre)2016-2019(post); surveys 6-8months after training	**Principles of engagement and design:**<br>• Involvement of senior staff may increase staff engagement in the training: Only 3 clinical champions were involved in the study, and they were not in leadership positions.<br>• The different components of this intervention complemented each other resulting in the success of this intervention.<br><br>**Considerations for improving healthcare systems:**<br>• Importance of using interpreters: increased interpreter uptake and improved patient outcome.<br>• The multicomponent intervention contributed to good outcomes. This included training and championing, as well as providing the mechanism to enable new behaviours (through a Coordinator)<br><br>**Considerations for improving healthcare workforce:**<br>• The Aboriginal Interpreter Coordinator worked across all wards of the hospital providing staff support: including provision of mentoring and education for interpreters on assignment to the hospital and helping them to navigate the hospital environment.<br>• Increase engagement of staff is needed: Only a small proportion of healthcare providers attended the working with interpreter training sessions.<br><br>**Sustainability of the intervention and outcomes:**<br>• Further strategies to improve the proportion of Indigenous patients accessing high-quality communication in this setting are required as a core strategy to improve health outcomes. To achieve the much greater-magnitude change required, substantial investment in combined approaches for upscaling interpreter use addressing supply, demand, efficiency and effectiveness, are needed.
Passey & Stirling	Mixed methods Demographic, obstetric and	19 pregnant women with a history of smoking – 17	Smoking cessation program: culturally tailored and included individual tailored counselling, free nicotine	**Duration:** Initial baseline visit followed by another follow-up 2-4 days later. Subsequently, visited twice	**Principles of engagement and design:**

Author (year) Location	Method and Study design	Study population	Brief description of the intervention	Duration of intervention & measurement period	Key learnings
(2018)[19] NSW	smoking behaviour. Interviews with pregnant women and staff.	Aboriginal women, 2 non-Aboriginal women with Aboriginal partners	replacement therapy, engagement with household members, specially developed resources, contingency-based financial rewards and peer support groups.	weekly at home for 3 weeks, weekly for 4 weeks, then fortnightly until the birth of their baby.<br><br>(>12 contacts) over at least 19 weeks<br><br>**Measurement period:** During the program (initial, late pregnancy) – no measurements after birth	• This program was resource intensive, future programs to consider resources available: Feasibility issues included challenges providing twice-weekly visits for 3 weeks and running fortnightly support groups.<br>• Rapport with women through the existing program was critical to engagement, so any additional health worker would need to be integrated into the team.<br><br>**Considerations for improving healthcare systems:**<br>• Intensive support for pregnant women trying to quit smoking was identified as a key factor to successful smoking cessation.<br><br>**Considerations for improving healthcare workforce:**<br>-none-<br><br>**Sustainability:**<br>• It is noted that there was a lack of capacity to sustain the program with existing resources.<br><br>• Unknown rates of abstinence from smoking after birth.
Prout et al. (2014)[20] WA	Qualitative Student journal entries	27 health students	An experiential and hands-on approach to education in rural health settings: students were placed in real rural health contexts.	**Duration:** 1 week placement, different students placed over 1 year<br><br>**Measurement period:** Assessing the outcomes was conducted at the beginning and end of the one-year timeframe.	**Principles of engagement and design:**<br>• Experiential and collaborative learning: The program emphasises hands-on experience and collaboration between students, educators, and community members to improve practical applications and share insights.<br>• Contextual Reflection and Empowerment: Participants were encouraged to reflect on their learning in the curriculum as required by Rural Health. Promotes personal growth and empower students to take initiative in their studies.<br><br>**Considerations for improving healthcare systems:**<br>• To increase the efficiency of training and service provision. healthcare systems should prioritise understanding rural health challenges. and promote interdisciplinary collaboration between health professionals and educators.<br>• Promoting reflective practice by facilitating hands-on learning can empower healthcare professionals. This will help improve job retention and satisfaction, treatment and overall patient care in rural areas.<br><br>**Considerations for improving healthcare workforce:**<br>• Rural health placements imparted skills that are not able to be taught in lecture-based courses.<br>• Active community contact to address real-life obstacles enriches learning and fosters a deeper comprehension of rural health issues.<br>• Enhanced knowledge of rural health issues and built confidence in practical situations through direct learning and reflection, which improved community

Author (year) Location	Method and Study design	Study population	Brief description of the intervention	Duration of intervention & measurement period	Key learnings
					linkages and increased their commitment to rural health practices, effectively preparing them for its challenges.<br><br>**Sustainability of the intervention and outcomes:**<br>• Sustainability depends on adaptive learning being embedded within the curriculum, institutional support, participation of both students and teachers, flexibility to meet rural health needs, and continuity in resources to effectively enhance the health education outcomes.
Rae et al. (2014)[21] NSW	Mixed methods Obstetric data, descriptive	100 pregnant Indigenous women, Indigenous attendees of the Gomeroi gaaynggal Centre	The Gomeroi gaaynggal program aimed at reducing renal disease in women and their children: an art group that meets weekly run by a local Indigenous artist and attended by health professionals, providing education to pregnant Indigenous women about topics related to renal disease.	**Duration:** This study started in 2009 and at the time of publication (2014) was ongoing<br><br>**Measurement period:** Each trimester of pregnancy; samples are taken from the mother and measurements of the foetus and foetal kidney. Post-natal longitudinal follow-up for an unspecified period of time	**Principles of engagement and design:**<br>• Taking time to build trust with the participants was important for engagement.<br>• Using person-centred goals and collaborative approach: Motivating participants through positive goal setting rather than giving authoritative directives.<br><br>**Considerations for improving healthcare systems:**<br>• Integrating art groups and health education can be an effective way of engaging Indigenous women<br><br>**Considerations for improving healthcare workforce:**<br>• An interdisciplinary approach, including artists, can help improve Indigenous health behaviours and assist with developing culturally appropriate health workforce through shared knowledge<br><br>**Sustainability of the intervention and outcomes:**<br>• Sustainable as long as funding continues
Ralph et al. (2018)[22] NT	Mixed methods Stepped-wedge randomised controlled trial Interviews	10 remote Indigenous community clinics 304 Indigenous patients with ARF and/or RDH requiring penicillin injections	Stepped-wedge, randomized trial: a multicomponent intervention supporting activities to improve penicillin delivery, aligned with the chronic care model, with continuous quality-improvement feedback on adherence.	**Duration:** 12-month baseline phase, 3-month transition phase, 12-month intensive phase and a 3- to 12-month maintenance phase.<br><br>**Measurement period:** 12-month baseline phase, 3-month transition phase, no post-intervention measurement.	**Principles of engagement and design:**<br>• Longer interventions are needed, with more community linkages, to achieve better adherence in this cross-cultural context.<br><br>**Considerations for improving healthcare systems:**<br>• Critical importance of improving engagement between healthcare services and Indigenous patients.<br><br>**Considerations for improving healthcare workforce:**<br>• Barriers to improving engagement of Indigenous patients need to be addressed by engaging the highest levels of health system using innovative staff education and retention measures and ensuring cultural security within healthcare environments.<br><br>**Sustainability:**

Author (year) Location	Method and Study design	Study population	Brief description of the intervention	Duration of intervention & measurement period	Key learnings
					• Patients who were already well engaged experienced benefit during longer term follow-up.
Read et al. (2018)[23] NT	Mixed methods Interviews, participant observation and project officer reports. Audit of action items	121 clinic staff, 22 key informants, 72 Indigenous patients with ARF and/or RHD and family members	Process evaluation of the intervention described in Ralph et al. (2018)'s paper.	**Duration:** As above in Ralph et al. (2018)'s paper.<br><br>**Measurement period:** "at the outset"	**Principles of engagement and design:**<br>• Health-system–strengthening activities at primary care centres fail to change outcomes for clients if strategies are insufficient to successfully engage the community and provide chronic disease self-management support.<br>• Consider language barriers and opportunities for client questions: Client interviews revealed that they often felt powerless to ask questions about their condition in the clinic environment.<br><br>**Considerations for improving healthcare systems:**<br>• Improving chronic condition management in primary care requires a comprehensive approach to chronic care management, especially to activities fostering client and community engagement.<br><br>**Considerations for improving healthcare workforce:**<br>-none-<br><br>**Sustainability of the intervention and outcomes:**<br>• Outcomes desired were not achieved. Barriers identified included project-level factors (short trial duration), implementation factors (types of activities implemented), and contextual factors (high staff turnover and the complex sociocultural environment).
Reeve et al. (2014)[24] WA	Quantitative Cross-sectional retrospective audit of medical records and referral letters	Baseline: 148 Indigenous children Post-program: 710 Indigenous children	Implementation of an ear health team in primary care to ensure high-quality referrals and a more patient-centred approach to continuity of care and follow up.	**Duration:** Systems changes – new processes intended as ongoing<br><br>**Measurement period:** Audits done prior to implementing intervention and 18 months later	**Principles of engagement and design:**<br>• Effective use of technology and resources: Patients that did not attend in person were discussed with the ENT specialist and due to availability of the otoscopic photo and diagnostic test results in the electronic referral, appropriate management was able to be arranged. The telehealth service increased efficiency.<br>• Effective implementation should be embedded in the system and not be reliant on a single person: A reduction in referrals and telehealth sessions occurred when the ear health nurse was on extended leave.<br><br>**Considerations for improving healthcare systems:**<br>• The ear health pathway allowed for more patients to be engaged with higher quality care: more referrals and improved quality of referrals to ENT specialists, and improved management including appropriate antibiotic use and follow up.<br>• Chronic disease and mortality can be reduced through implementation of an ear health team in primary care settings.

Author (year) Location	Method and Study design	Study population	Brief description of the intervention	Duration of intervention & measurement period	Key learnings
					• The ear health pathway decreased wait time: 78% of cases were reviewed via the Telehealth service with a decrease in wait time despite the increased number of referrals.<br><br>**Considerations for improving healthcare workforce:**<br>• The study recommended training of Indigenous project officers by the ear health educator and nurse to take on roles to improve sustainability of the service.<br><br>**Sustainability of the intervention and outcomes:**<br>• Sustainability relies upon changes in the system with a multicomponent intervention; appeared well embedded within the study period
Reeve et al. (2015)[25] WA	Quantitative Cross-sectional retrospective evaluation of health service data	2006: 2160 individuals on electronic health record<br>2012: 5410 individuals on electronic health record	Evaluation of the impact of integration of policy implementation, health promotion, health assessments and chronic disease management between an acute primary health care service through formal partnership between the hospital, community health service and community controlled health service.	**Duration:** 6 years retrospective evaluation<br><br>**Measurement period:** July 2006 – June 2012	**Principles of engagement and design:**<br>• Evaluation of a partnership between health services to reorient them away from an existing acute reactive approach to a more comprehensive primary health care approach in line with recommendations from the National Strategies for Improving Indigenous Health and Health Care<br><br>**Considerations for improving healthcare systems:**<br>• Using a primary health care model as opposed to a reactive acute approach would result in improved primary health for the population. Outcomes translated into health gains consistent with goals of the National Health Care Reform and Closing the Gap policies and may potentially reduce health inequity for remote-living Indigenous Australians.<br>• Outcomes included decrease in number of deaths during the 6 years, increase in screening for alcohol and tobacco use and a decrease in percentage of hospital admissions requiring emergency evacuation.<br><br>**Considerations for improving healthcare workforce:**<br>• Recommendations from the community controlled health service led to increased employment of Indigenous staff and cultural training for all staff which in turn led to a better understanding of the importance of families and guardianship roles, including traditional healers.<br><br>**Sustainability of the intervention and outcomes:**<br>• Embedded into systems: Original intervention was begun with a Memorandum of Understanding between the three health services and then various changes to funding and ability to claim on Medicare services.

Author (year) Location	Method and Study design	Study population	Brief description of the intervention	Duration of intervention & measurement period	Key learnings
Schoen et al. (2016)[26] WA	Quantitative Quasi-experimental, pre-test/post-test survey	246 rural and remote health professionals	Education to enhance rural and remote practitioners' understanding of diabetic foot care.	**Duration:** 1x 3h workshop<br><br>**Measurement period:** pre-intervention assessments, immediate post-intervention evaluations on the same day	**Principles of engagement and design:**<br>• Adaptations made the program more acceptable and appropriate for Indigenous people.<br><br>**Considerations for improving healthcare systems:**<br>• The interdisciplinary approach encouraged collaboration between different healthcare providers, leading to the development of a comprehensive care plan for patients with diabetic foot problems.<br><br>**Considerations for improving healthcare workforce:**<br>• Practical, hands-on workshops were essential, allowing practitioners to apply what they learned in a controlled environment, improving their skills and confidence.<br>• Regular follow-up sessions are recommended to help reinforce knowledge and skills.<br>• Geographical isolation of some rural and remote areas made it difficult to ensure consistent training<br><br>**Sustainability of the intervention and outcomes:**<br>• Follow-up support to reinforce new knowledge was lacking, resulting in minimal long-term impact on practitioners' practice and patient outcomes.
Shephard et al. (2016)[27] National	Mixed methods Focus group discussion Survey	4 members of the QAAMS Indigenous Leaders Team 104 QAAMS POCT operators	Point-of-care pathology testing for diabetes management	**Duration:** Service implementation since 2004, ongoing<br><br>**Measurement period:** 10 years since initial survey about cultural safety	**Principles of engagement and design:**<br>• Build relationships and trust with communities and stakeholders<br>• Ensure adequate staffing and resourcing – it was a challenge in this study<br><br>**Considerations for improving healthcare systems:**<br>• Take the opportunity to deliver health promotion through yarning with patients while waiting for test results.<br>• Point of care testing increases engagement of patients as it increases the efficiency of services<br><br>**Considerations for improving healthcare workforce:**<br>• Culturally effective training for staff was important<br><br>**Sustainability of the intervention and outcomes:**<br>-none-
Spaeth & Shephard	Mixed methods Evaluation of health service	32 remote health services conduct	Point-of-care testing to monitor the efficacy of warfarin therapy in	**Duration:** Since 2008, ongoing<br><br>**Measurement period:**	**Principles of engagement and design:**<br>-none-<br><br>**Considerations for improving healthcare systems:**

Author (year) Location	Method and Study design	Study population	Brief description of the intervention	Duration of intervention & measurement period	Key learnings
(2016)[28] NT	data<br>Case studies	INR POCT<br>4 case studies	rheumatic heart disease management	2008 - 2014	• Point of care testing increases engagement of patients as it increases the efficiency of services, and increase in the number of patients accessing the tests<br><br>**Considerations for improving healthcare workforce:**<br>-none-<br><br>**Sustainability of the intervention and outcomes:**<br>• "The main challenges for the program's sustainability continue to be the current lack of Medicare reimbursement from the Australian Government for the INR test on the i-STAT device."
Thackrah & Thompson (2013a)[29] WA	Mixed methods<br>Questionnaires and classroom observations	12 non-Indigenous midwifery students	University undergraduate tutorials about cultural safety and security for health science students: Aboriginal and Torres Strait Islander history, diversity, cultural protocols, social structures, patterns of communication, contemporary policies and their implications for health professionals.	**Duration:**<br>One university semester (2 hours per week, over 5 months)<br><br>**Measurement period:**<br>Post-unit completion (unspecified time)	**Principles of engagement and design:**<br>• Team teaching may be limited by resource availability.<br>• Consistent attendance over the semester of an observer using an 'complete observer' approach was an effective way of observing classroom behaviours – the researcher had become almost invisible.<br>• Involvement of Indigenous tutors was important; when students had the opportunity to interact with Indigenous people in a classroom environment, pre-existing stereotypes were challenged in a way that was different to that achieved by watching excerpts on film.<br><br>**Considerations for improving healthcare systems:**<br>• Education about Indigenous health can start during university training in health courses, to create more culturally secure services as students progress into the workforce.<br><br>**Considerations for improving healthcare workforce:**<br>• Importance of creating a safe learning environment for opinions to be discussed, including the ability of facilitators to manage distress of participants when controversial discussions occur<br>• Students wanted more cultural knowledge about their specific area of practice (pregnancy and birthing for midwife students)<br><br>**Sustainability of the intervention and outcomes:**<br>• Unknown if students continue to advocate for cultural security when they transition to the workforce.
Thackrah & Thompson (2013b)[30] WA	Mixed methods<br>Questionnaire and interviews	15 midwifery students	University undergraduate tutorials about cultural safety and security for health science students: Aboriginal and Torres Strait Islander	**Duration:**<br>One university semester (2 hours per week)<br><br>**Measurement period:**	**Principles of engagement and design:**<br>• Consider the safety of Indigenous students in the group, especially when racist comments are made by other students during discussions.<br><br>**Considerations for improving healthcare systems:**

Author (year) Location	Method and Study design	Study population	Brief description of the intervention	Duration of intervention & measurement period	Key learnings
			history, diversity, cultural protocols, social structures, patterns of communication, contemporary policies and their implications for health professionals.<br>(as above)	Post-unit completion (unspecified time)	• Education about Indigenous health can start during university training in health courses, to create more culturally secure services as students progress into the workforce.<br><br>**Considerations for improving healthcare workforce:**<br>• Smaller class sizes are more effective for change. Large lecture theatres usually give students anonymity and less participation.<br>• Overcoming some of the challenges, such as addressing racism and having difficult conversations that evoke uncomfortable emotions is beneficial in the education about Indigenous health and determinants.<br><br>**Sustainability of the intervention and outcomes:**<br>-none-
Thackrah et al. (2015a)[31] WA	Quantitative Survey	44 students in a direct entry, undergraduate midwifery program	University undergraduate tutorials about cultural safety and security for health science students: Aboriginal and Torres Strait Islander history, diversity, cultural protocols, social structures, patterns of communication, contemporary policies and their implications for health professionals.<br>(as above)	**Duration:**<br>One university semester (2 hours per week)<br><br>**Measurement period:**<br>First year students: Pre-unit and post-unit completion,<br><br>Second- and Third-year students: 1-2 years after unit completion	**Principles of engagement and design:**<br>• Consider timing of unit/continuation of education over the years: vertical integration of Indigenous content in curricula was suggested.<br><br>**Considerations for improving healthcare systems:**<br>• Education about Indigenous health can start during university training in health courses, to create more culturally secure services as students progress into the workforce.<br><br>**Considerations for improving healthcare workforce:**<br>• Provide more information on Indigenous cultural protocols surrounding pregnancy, birth and the post-partum period for midwifery students.<br>• The provision of more clinical practice opportunities with Indigenous women is likely to strengthen culturally secure care.<br><br>**Sustainability of the intervention and outcomes:**<br>• Immediate increase in confidence in interactions: Pre- and post-unit changes were observed with respect to students' perceptions of their capacity to communicate with Indigenous women.<br>• Inadequate continuation of confidence: 25% of the second- and third-year students considered their knowledge about Indigenous health to be inadequate – need for ongoing education.
Thackrah et al. (2015b)[32] WA	Qualitative Interviews	7 midwifery students	A short rural clinical placement for midwifery students focused on antenatal care, sexual health, breast	**Duration:**<br>Up to 2 weeks<br><br>**Measurement period:**	**Principles of engagement and design:**<br>• Importance of incorporating student self-reflections during the learning process.<br><br>**Considerations for improving healthcare systems:**

Author (year) Location	Method and Study design	Study population	Brief description of the intervention	Duration of intervention & measurement period	Key learnings
			screening and promotion of healthy behaviours	Unspecified (ranged from months to years)	• Education about Indigenous health can start during university training in health courses, to create more culturally secure services as students progress into the workforce.<br><br>**Considerations for improving healthcare workforce:**<br>• Importance of including delivery of services on-Land during training: exposed students to hands-on learning and appreciation for issues that are unique to rural areas.<br>• Importance of in-person experiences with Indigenous communities during training: allowed for learning of culturally appropriate clinical skills, community needs, and building confidence.<br>• Enhanced health awareness and practices among Indigenous women, coupled with increased cultural competence among midwifery students, can lead to more effective, tailored health interventions that promote long-term health benefits for these communities.<br><br>**Sustainability of the intervention and outcomes:**<br>• Sustainability of placements depend on universities providing opportunities, and community practitioner's willingness to accept students.
Tsey et al. (2014)[33] Multiple states	Qualitative Interviews, workshops, focus groups and participant observations	25 participants - 9 Male Health Module trainers, 16 health workers who attended the Male Health Module workshop	An educational module about Aboriginal and Torres Strait Islander male health: 15 units across a range of health areas	**Duration:** Unspecified (the module was still in development phase in this pilot study)<br><br>**Measurement period:** At the end of each pilot workshop	**Principles of engagement and design:**<br>• Importance of broadness of scope in male health: to include rural and remote communities, youth engagement strategies, and those with limited access to health service facilities, while tailoring delivery to meet the unique needs of each age group or community.<br>• Integrate with existing health programs: Involve local health organisations, community groups and government agencies in incorporating the module into existing health programs<br>• Positive changes in health knowledge and behaviours among participants, lead to improved health literacy, increased awareness of health services, and greater confidence in managing their health.<br>• The long-term impact of the project is supported by community engagement and culturally relevant practices; promoting local ownership and commitment.<br><br>**Considerations for improving healthcare systems:**<br>• Improve monitoring and evaluation to inform future versions of services and capacity building for healthcare workers.<br><br>**Considerations for improving healthcare workforce:**<br>• Increase Cultural Competency Training: Develop and deliver ongoing cultural competency training for health workers, incorporating Indigenous health workers and leaders, while also creating resources that showcase successful practices in understanding Indigenous cultures and health beliefs.

Author (year) Location	Method and Study design	Study population	Brief description of the intervention	Duration of intervention & measurement period	Key learnings
					• Continuous professional development: providing access to resources, support networks, and opportunities for peer-to-peer learning and mentoring to effectively address complex health issues.<br><br>**Sustainability of the intervention and outcomes:**<br>-none-

Abbreviations: ABCD = audit and best practice in chronic disease; AHP = Aboriginal Health Practitioner; AHW = Aboriginal Health Worker; AMS = Aboriginal Medical Service; ARF = acute rheumatic fever; CQI = clinical quality improvement; DAHAG = District Aboriginal Health Action Group; ENT = ear, nose and throat; INR = international normalized ratio; LBP = lower back pain; LWSF = Living Well Smoke Free; NSW = New South Wales; NT = Northern Territory; POCT = point-of-care testing; QAAMS = Quality Assurance for Aboriginal and Torres Strait Islander Medical Services; QLD = Queensland; RHD = rheumatic heart disease; Vic = Victoria; WA = Western Australia

References

1. Bailie, R.; Matthews, V.; Larkins, S.; Thompson, S.; Burgess, P.; Weeramanthri, T.; Bailie, J.; Cunningham, F.; Kwedza, R.; Clark, L. Impact of policy support on uptake of evidence-based continuous quality improvement activities and the quality of care for Indigenous Australians: a comparative case study. *BMJ open* **2017**, *7*, e016626, doi:10.1136/bmjopen-2017-016626.
2. Bennett-Levy, J.; Singer, J.; DuBois, S.; Hyde, K. Translating E-Mental Health Into Practice: What Are the Barriers and Enablers to E-Mental Health Implementation by Aboriginal and Torres Strait Islander Health Professionals? *J Med Internet Res* **2017**, *19*, e1, doi:10.2196/jmir.6269.
3. Bennett-Levy, J.; Singer, J.; Rotumah, D.; Bernays, S.; Edwards, D. From Digital Mental Health to Digital Social and Emotional Wellbeing: How Indigenous Community-Based Participatory Research Influenced the Australian Government's Digital Mental Health Agenda. *International Journal of Environmental Research and Public Health* **2021**, *18*, 9757, doi:10.3390/ijerph18189757.
4. Biles, J.; Deravin, L.; Seaman, C.E.; Alexander, N.; Damm, A.; Trudgett, N. Learnings from a mentoring project to support Aboriginal and Torres Strait Islander nurses and midwives to remain in the workforce. *Contemporary nurse* **2021**, *57*, 327-337, doi:10.1080/10376178.2021.1991412.
5. Brimblecombe, J.; Ferguson, M.; Chatfield, M.D.; Liberato, S.C.; Gunther, A.; Ball, K.; Moodie, M.; Miles, E.; Magnus, A.; Mhurchu, C.N.; et al. Effect of a price discount and consumer education strategy on food and beverage purchases in remote Indigenous Australia: a stepped-wedge randomised controlled trial. *Lancet Public Health* **2017**, *2*, e82-e95, doi:10.1016/S2468-2667(16)30043-3.

6. Cairns, A.; Geia, L.; Kris, S.; Armstrong, E.; O'Hara, A.; Rodda, D.; McDermott, R.; Barker, R. Developing a community rehabilitation and lifestyle service for a remote indigenous community. *Disability and Rehabilitation* **2022**, *44*, 4266-4274, doi:10.1080/09638288.2021.1900416.
7. Carey, T.A.; Schouten, K.; Wakerman, J.; Humphreys, J.S.; Miegel, F.; Murphy, S.; Arundell, M. Improving the quality of life of palliative and chronic disease patients and carers in remote Australia with the establishment of a day respite facility. *BMC Palliative Care* **2016**, *15*, 62, doi:10.1186/s12904-016-0136-1.
8. Chapple, K.; Kowanko, I.; Harvey, P.; Chong, A.; Battersby, M. 'Imagine if I gave up smoking ...': a qualitative exploration of Aboriginal participants' perspectives of a self-management pilot training intervention. *Australian Journal of Primary Health* **2016**, *22*, 147-152, doi:10.1071/PY14104.
9. Durey, A.; McEvoy, S.; Swift-Otero, V.; Taylor, K.; Katzenellenbogen, J.; Bessarab, D. Improving healthcare for Aboriginal Australians through effective engagement between community and health services. *BMC Health Services Research* **2016**, *16*, 224, doi:10.1186/s12913-016-1497-0.
10. Fernando, S.; Tadakamadla, S.; Kroon, J.; Lalloo, R.; Johnson, N.W. Predicting dental caries increment using salivary biomarkers in a remote Indigenous Australian child population. *BMC Oral Health* **2021**, *21*, 372, doi:10.1186/s12903-021-01702-0.
11. Guy, R.J.; Ward, J.; Causer, L.M.; Natoli, L.; Badman, S.G.; Tangey, A.; Hengel, B.; Wand, H.; Whiley, D.; Tabrizi, S.N.; et al. Molecular point-of-care testing for chlamydia and gonorrhoea in Indigenous Australians attending remote primary health services (TTANGO): a cluster-randomised, controlled, crossover trial. *Lancet Infect Dis* **2018**, *18*, 1117-1126, doi:10.1016/S1473-3099(18)30429-8.
12. Haigh, M.; Shahid, S.; O'Connor, K.; Thompson, S.C. Talking about the not talked about: use of, and reactions to, a DVD promoting bowel cancer screening to Aboriginal people. *Australian and New Zealand journal of public health* **2016**, *40*, 548-552, doi:10.1111/1753-6405.12565.
13. Isaacs, A.; Lampitt, B. The Koorie Men's Health Day: an innovative model for early detection of mental illness among rural Aboriginal men. *Australasian Psychiatry* **2014**, *22*, 56-61, doi:10.1177/1039856213502241.
14. Khalil, H. Successful implementation of a medication safety program for Aboriginal Health Practitioners in rural Australia. *Australian Journal of Rural Health* **2019**, *27*, 158-163, doi:10.1111/ajr.12494.
15. Kong, A.; Dickson, M.; Ramjan, L.; Sousa, M.S.; Jones, N.; Srinivas, R.; Chao, J.; Goulding, J.; George, A. Aboriginal Health Workers Promoting Oral Health among Aboriginal and Torres Strait Islander Women during Pregnancy: Development and Pilot Testing of the Grinnin' Up Mums & Bubs Program. *International Journal of Environmental Research and Public Health* **2021**, *18*, 9576, doi:10.3390/ijerph18189576.
16. Lalloo, R.; Tadakamadla, S.K.; Kroon, J.; Jamieson, L.M.; Ware, R.S.; Johnson, N.W. Carious lesions in permanent dentitions are reduced in remote Indigenous Australian children taking part in a non-randomised preventive trial. *PloS one* **2021**, *16*, e0244927, doi:10.1371/journal.pone.0244927.

17. Lin, I.B.; Coffin, J.; O'Sullivan, P.B. Using theory to improve low back pain care in Australian Aboriginal primary care: a mixed method single cohort pilot study. *BMC Family Practice* **2016**, *17*, 44, doi:10.1186/s12875-016-0441-z.
18. O'Connor, E.; Kerrigan, V.; Aitken, R.; Castillon, C.; Mithen, V.; Madrill, G.; Roman, C.; Ralph, A.P. Does improved interpreter uptake reduce self-discharge rates in hospitalised patients? A successful hospital intervention explained. *PloS one* **2021**, *16*, e0257825, doi:10.1371/journal.pone.0257825.
19. Passey, M.E.; Stirling, J.M. Evaluation of 'Stop Smoking in its Tracks': an intensive smoking cessation program for pregnant Aboriginal women incorporating contingency-based financial rewards. *Public Health Research & Practice* **2018**, *28*, e28011804, doi:10.17061/phrp28011804.
20. Prout, S.; Lin, I.; Nattabi, B.; Green, C. 'I could never have learned this in a lecture': transformative learning in rural health education. *Advances in Health Sciences Education: Theory and Practice* **2014**, *19*, 147-159, doi:10.1007/s10459-013-9467-3.
21. Rae, K.; Weatherall, L.; Blackwell, C.; Pringle, K.; Smith, R.; Lumbers, E. Long conversations: Gomeroi gaaynggal tackles renal disease in the Indigenous community. *Australasian Epidemiologist* **2014**, *21*, 44-48, doi:10.3316/informit.332850582191092.
22. Ralph, A.P.; de Dassel, J.L.; Kirby, A.; Read, C.; Mitchell, A.G.; Maguire, G.P.; Currie, B.J.; Bailie, R.S.; Johnston, V.; Carapetis, J.R. Improving Delivery of Secondary Prophylaxis for Rheumatic Heart Disease in a High-Burden Setting: Outcome of a Stepped-Wedge, Community, Randomized Trial. *Journal of the American Heart Association* **2018**, *7*, e009308, doi:10.1161/JAHA.118.009308.
23. Read, C.; Mitchell, A.G.; de Dassel, J.L.; Scrine, C.; Hendrickx, D.; Bailie, R.S.; Johnston, V.; Maguire, G.P.; Schultz, R.; Carapetis, J.R.; et al. Qualitative Evaluation of a Complex Intervention to Improve Rheumatic Heart Disease Secondary Prophylaxis. *Journal of the American Heart Association* **2018**, *7*, e009376, doi:10.1161/JAHA.118.009376.
24. Reeve, C.; Thomas, A.; Mossenson, A.; Reeve, D.; Davis, S. Evaluation of an ear health pathway in remote communities: improvements in ear health access. *Australian Journal of Rural Health* **2014**, *22*, 127-132, doi:10.1111/ajr.12098.
25. Reeve, C.; Humphreys, J.; Wakerman, J.; Carter, M.; Carroll, V.; Reeve, D. Strengthening primary health care: achieving health gains in a remote region of Australia. *Medical Journal of Australia* **2015**, *202*, 483-487, doi:10.5694/mja14.00894.
26. Schoen, D.E.; Gausia, K.; Glance, D.G.; Thompson, S.C. Improving rural and remote practitioners' knowledge of the diabetic foot: findings from an educational intervention. *Journal of Foot and Ankle Research* **2016**, *9*, 26, doi:10.1186/s13047-016-0157-2.
27. Shephard, M.; O'Brien, C.; Burgoyne, A.; Croft, J.; Garlett, T.; Barancek, K.; Halls, H.; McAteer, B.; Motta, L.; Shephard, A. Review of the cultural safety of a national Indigenous point-of-care testing program for diabetes management. *Australian journal of primary health* **2016**, *22*, 368-374, doi:10.1071/PY15050.
28. Spaeth, B.A.; Shephard, M.D.S. Clinical and Operational Benefits of International Normalized Ratio Point-of-Care Testing in Remote Indigenous Communities in Australia's Northern Territory. *Point of Care* **2016**, *15*, 30-34, doi:10.1097/poc.0000000000000082.
29. Thackrah, R.D.; Thompson, S.C. Confronting uncomfortable truths: receptivity and resistance to Aboriginal content in midwifery education. *Contemporary nurse* **2013**, *46*, 113-122, doi:10.5172/conu.2013.46.1.113.

30. Thackrah, R.D.; Thompson, S.C. 'Friendly racism' and white guilt: midwifery students' engagement with Aboriginal content in their program. *Forum on Public Policy* **2013**, *2013*, 12.
31. Thackrah, R.D.; Thompson, S.C.; Durey, A. Exploring undergraduate midwifery students' readiness to deliver culturally secure care for pregnant and birthing Aboriginal women. *BMC Medical Education* **2015**, *15*, 77, doi:10.1186/s12909-015-0360-z.
32. Thackrah, R.D.; Thompson, S.C.; Durey, A. Promoting women's health in remote Aboriginal settings: Midwifery students' insights for practice. *Australian Journal of Rural Health* **2015**, *23*, 327-331, doi:10.1111/ajr.12247.
33. Tsey, K.; Chigeza, P.; Holden, C.A.; Bulman, J.; Gruis, H.; Wenitong, M. Evaluation of the pilot phase of an Aboriginal and Torres Strait Islander Male Health Module. *Australian Journal of Primary Health* **2014**, *20*, 56-61, doi:10.1071/PY12033.
